# Doxorubicin-Based Chemotherapy for the Palliative Treatment of Adult Patients with Locally Advanced or Metastatic Soft-Tissue Sarcoma: A Meta-Analysis and Clinical Practice Guideline

**DOI:** 10.1080/13577140020008066

**Published:** 2000-09

**Authors:** Vivien H. C. Bramwell, Dale Anderson, Manya L. Charette

**Affiliations:** 1 London Regional Cancer Centre London Ontario Canada; 2 Cancer Care Ontario Program in Evidence-based Care Hamilton Ontario Canada

## Abstract

*Purpose.* To make recommendations for the use of doxorubicin-based chemotherapy in patients with soft-tissue sarcoma.

*Patients.* The recommendations apply to patients with symptomatic unresectable locally advanced or metastatic soft-tissue sarcoma who are candidates for palliative chemotherapy.

*Methods.* A systematic review of the published literature was combined with a consensus process around the interpretation of the evidence in the context of conventional practice to develop an evidence-based practice guideline.

*Results.* Eight randomized trials comparing doxorubicin-based combination versus doxorubicin single-agent chemotherapy were reviewed. Response rates and overall survival were evaluated using pooled statistical analysis.The pooled response data in 2281 patients showed a slight trend favouring the combination therapy, although this did not reach statistical significance (odds ratio (OR), 0.79; 95% confidence interval (CI), 0.60–1.05; *p*=0.10). Survival data could only be abstracted from six studies involving 2097 patients, and showed no significant advantage for combination therapy (OR, 0.84; 95% CI, 0.67–1.06; *p*=0.13). Data on adverse effects could not be combined in a meta-analysis; however nausea, vomiting and myelosuppression were consistently more severe with combination chemotherapy than with single-agent chemotherapy.

*Discussion.* Single-agent doxorubicin is an appropriate first-line chemotherapy option for advanced or metastatic soft-tissue sarcoma. Some doxorubicin-based combination chemotherapy regimens, given in conventional doses, produce only marginal increases in response rates, at the expense of increased adverse effects, and with no improvements in overall survival. Future randomized clinical trials should compare new regimens, whose activity has been established in single-arm studies, with single-agent doxorubicin, and include quality of life as an outcome measure.


Sarcoma (2000) 4, 103–112

ORIGINAL ARTICLE

### Doxorubicin-based chemotherapy for the palliative treatment of adult patients with locally advanced or metastatic soft-tissue sarcoma: a meta-analysis and clinical practice guideline

VIVIEN H.C. BRAMWELL[1], DALE ANDERSON[2], MANYA L. CHARETTE[2] & THE MEMBERS OF THE CANCER CARE ONTARIO PRACTICE GUIDELINES INITIATIVE SARCOMA DISEASE SITE GROUP

*[1]London Regional Cancer Centre, London, Ontario, [2]Cancer Care Ontario Program in Evidence-based Care, Hamilton, Ontario, Canada*

**Abstract**
*Purpose*. To make recommendations for the use of doxorubicin-based chemotherapy in patients with soft-tissue sarcoma.
*Patients*. The recommendations apply to patients with symptomatic unresectable locally advanced or metastatic soft-tissue sarcoma who are candidates for palliative chemotherapy.
*Methods*. A systematic review of the published literature was combined with a consensus process around the interpretation of the evidence in the context of conventional practice to develop an evidence-based practice guideline.
*Results*. Eight randomized trials comparing doxorubicin-based combination versus doxorubicin single-agent chemotherapy were reviewed. Response rates and overall survival were evaluated using pooled statistical analysis. The pooled response data in 2281 patients showed a slight trend favouring the combination therapy, although this did not reach statistical significance (odds ratio (OR), 0.79; 95% confidence interval (CI), 0.60–1.05; $p$=0.10). Survival data could only be abstracted from six studies involving 2097 patients, and showed no significant advantage for combination therapy (OR, 0.84; 95% CI, 0.67–1.06; $p$=0.13). Data on adverse effects could not be combined in a meta-analysis; however nausea, vomiting and myelosuppression were consistently more severe with combination chemotherapy than with single-agent chemotherapy.
*Discussion*. Single-agent doxorubicin is an appropriate first-line chemotherapy option for advanced or metastatic soft-tissue sarcoma. Some doxorubicin-based combination chemotherapy regimens, given in conventional doses, produce only marginal increases in response rates, at the expense of increased adverse effects, and with no improvements in overall survival. Future randomized clinical trials should compare new regimens, whose activity has been established in single-arm studies, with single-agent doxorubicin, and include quality of life as an outcome measure.

**Key words**: *soft-tissue sarcoma, doxorubicin, chemotherapy, practice guideline, meta-analysis*

### Introduction

Doxorubicin was first identified as an active agent in the treatment of adult soft-tissue sarcomas in the 1970s, and response rates in early studies ranged from 9% to 70%.[1] More recently, large randomized multi-centre studies have established response rates in the range of 16–27% for single-bolus doses of doxorubicin given every 3 weeks.[2–9] Subsequently, dacarbazine (DTIC) and ifosfamide were identified as active agents, with single-agent response rates of 18% for dacarbazine,[10] and 18–36% for ifosfamide.[11–13] A large number of other evaluated drugs have been shown to have minimal or inconsistent activity in patients with soft-tissue sarcomas.[14]

Various combinations of the active drugs have been evaluated in a number of non-randomized studies,[14,15] with documented response rates in the range of 35–60%, but generally at the expense of greater toxicity. Combination chemotherapy regimens not containing doxorubicin have consistently yielded poor results in adult patients with advanced soft-tissue sarcoma.[3,16,17] Results from large randomized studies,[2–9] comparing doxorubicin-based combination chemotherapy regimens with single-agent doxorubicin, have been more varied. In some of these trials, response rates have been higher in the combination chemotherapy arms, whereas in others primary outcomes have not been significantly different between the treatments.[4,7,9]

Thus, there is considerable controversy as to whether any added benefit of combination chemotherapy outweighs increased toxic effects and

Correspondence to: Dr Vivien Bramwell, Chair, Sarcoma Disease Site Group, London Regional Cancer Centre, 790 Commissioners Road East, London, Ontario N6A 4L6, Canada. Fax: (519) 685-8624; E-mail: vivien.bramwell@lrcc.on.ca

1357-714Xprint/1369-1643online/00/030103-10 © 2000 Cancer Care Ontario's Program in Evidence-based Care
*DOI: 10.1080/13577140020008066*

inconvenience to patients, as well as additional costs to health care systems. This has led to a substantial variation in clinical practice. The Sarcoma Disease Site Group (DSG) felt that a practice guideline, based on an unbiased, systematic review of the evidence, was warranted. The guideline was developed specifically to answer the following questions.

1. Is there an advantage, in terms of response rate or survival, in using doxorubicin-based combination chemotherapy compared with single-agent doxorubicin for the palliative treatment of patients with incurable locally advanced or metastatic soft-tissue sarcoma?
2. Is combination chemotherapy associated with increased toxic effects compared with single-agent doxorubicin in this setting?

### Patients

This practice guideline addressed the treatment of patients with locally advanced or metastatic soft-tissue sarcoma who are candidates for palliative chemotherapy. Some patients with locally advanced soft-tissue sarcomas may be surgical candidates, and multi-disciplinary consultation between a specialized sarcoma surgeon, a radiation oncologist, a medical oncologist, a radiologist and a pathologist should be undertaken to determine the optimal management of these cases. A selected group of patients with metastases confined to the lungs (and rarely at other sites) may be suitable for resection with curative intent,[18,19] and this option should be considered prior to the use of palliative chemotherapy.

### Methods

### *Literature search strategy*

MEDLINE (Ovid) (from 1966) and CANCERLIT (Ovid) (from 1975) were searched in December 1997. 'Doxorubicin' (MeSH term and text word) was combined with 'combin' (truncated text word) and 'sarcoma' (MeSH term and text word), and these terms were then combined with search terms for the following study designs: practice guidelines; systematic reviews or meta-analysis; and randomized controlled trials. This search was updated in April and December 1998, June 1999 and January 2000. EMBASE was also searched from 1979 to 1995 using the truncated keywords 'random' and 'sarcoma'. Citation lists and personal files were scanned for additional studies. In addition, the *Proceedings of the Annual Meeting of the American Society of Clinical Oncology* (1995–99), and the Cochrane Library (issue 4, 1999), were also searched for additional reports of newly completed trials. No further attempt was made to find reports of unpublished randomized controlled trials. Relevant articles and abstracts were selected and assessed by two reviewers and the reference lists from these sources were searched for additional trials.

### *Study selection*

For a study to be eligible, it had to be a randomized controlled trial comparing single-agent doxorubicin with a doxorubicin-based combination chemotherapy regimen, and involve adult patients with locally advanced or metastatic soft-tissue sarcoma in the palliative setting. Potential studies had to measure response rate, overall survival and toxic effects or quality of life.

### *Pooling of trial results*

The intent was to combine (i.e. pool) data from all eligible trials, in order to calculate overall estimates of treatment efficacy and harm. Pooled results were expressed as an odds ratio (OR), which is the odds of an event occurring in the experimental group over the odds of an event occurring in the control group, with a 95% confidence interval (CI). Target events were consistently unfavourable (e.g. death at 2 years, no complete or partial response, etc.), so that estimates greater than 1.0 favoured the control group (single-agent therapy) and estimates less than 1.0 favoured the experimental group (combination therapy). The more conservative random effects model was used in the meta-analyses to allow for the differences in trial design and quality.[20] A statistical $Q$-test was used to measure the quantitative heterogeneity among study results. Calculations for the meta-analysis were performed on a Pentium PC using the software program Metaanalyst$^{0.988}$, created by Dr Joseph Lau (Boston, MA).

### *Guideline development process*

The guideline was developed out of the Cancer Care Ontario Practice Guidelines Initiative, using the methodology of the Practice Guidelines Development Cycle by Browman *et al.*[21] The guideline is a convenient and up-to-date source of the best available evidence on the use of doxorubicin-based chemotherapy for the palliative treatment of locally advanced or metastatic soft-tissue sarcoma. It has been developed through systematic reviews, evidence synthesis and input from practitioners in Ontario, Canada. It is intended to enable evidence-based practice.

Practitioner feedback was obtained through a mailed survey consisting of questions asking for ratings on the quality of the evidence-based recommendation (EBR) and whether the EBR should serve as a practice guideline.

### Results

### *Literature search results*

There were eight randomized controlled trials identified which met the eligibility criteria,[2–9] comparing doxorubicin combination chemotherapy with single-agent doxorubicin. Trial characteristics, including the chemotherapy regimens, are shown in Table 1. Outcome measures across all eight trials included response rates, median survival and various measures of toxicity, and these are shown in Tables 2 and 3. Response duration and time-to-progression were not reported consistently across studies and could not be analysed further. There were no practice guidelines or systematic reviews identified in the literature search.

**Table 1**. *Randomized controlled trials of doxorubicin combination chemotherapy in adult patients with incurable locally advanced or metastatic soft-tissue sarcoma*

| Study | Tumour type | Chemotherapy | Regimens* | Randomized patients (evaluable)† |
|---|---|---|---|---|
| Chang & Wiernik,[2] 1976, NCI (USA) | Adult STS (4 bone sarcomas) | DOX | 60 mg/m$^2$ IV bolus | 18 (17) |
| | 4 prior chemo | DOX<br>STREPT | 60 mg/m$^2$ IV bolus<br>500 mg/m$^2$ IV bolus days 1–5 | 15 (14) |
| Schoenfeld *et al.*,[3] 1982†, ECOG | Adult STS (18 bone | DOX | 70 mg/m$^2$ IV bolus | 71 (66) |
| | sarcomas, 9 mesotheliomas) 3 prior chemo | DOX<br>VCR<br>CYCLO | 50 mg/m$^2$ IV bolus<br>1.4 mg/m$^2$ IV bolus<br>750 mg/m$^2$ IV bolus | 80 (70) |
| Omura *et al.*,[4] 1983, GOG | Uterine sarcomas | DOX | 60 mg/m$^2$ IV bolus | 155 (120) |
| | 31 prior chemo | DOX<br>DTIC | 60 mg/m$^2$ IV bolus<br>250 m/m$^2$ IV bolus days 1–5 | 160 (106) |
| Muss *et al.*,[5] 1985, GOG | Uterine sarcomas | DOX | 60 mg/m$^2$ IV bolus | 66 (50) |
| | | DOX<br>CYCLO | 60 mg/m$^2$ IV bolus<br>500 mg/m$^2$ IV bolus | 66 (54) |
| Borden *et al.*,[6] 1987, ECOG | Adult STS | DOX | 70 mg/m$^2$ IV bolus | 123 (94) |
| | | DOX | 20 mg/m$^2$ days 1–3 IV bolus then 15 mg/m$^2$/week | 119 (88) |
| | | DOX<br>DTIC | 60 mg/m$^2$ IV bolus<br>250 mg/m$^2$ IV bolus days 1–5 | 119 (92) |
| Borden *et al.*,[7] 1990, ECOG | Adult STS | DOX | 70 mg/m$^2$ IV bolus | 176 (151) |
| | | DOX<br>VND | 70 mg/m$^2$ IV bolus<br>3 mg/m$^2$ IV bolus | 171 (147) |
| Edmonson *et al.*,[8] 1993, ECOG | Adult STS | DOX | 80 mg/m$^2$ IV bolus | 95 (90) |
| | (4 bone sarcomas) | DOX<br>IFOS | 60 mg/m$^2$ IV bolus<br>3.75 g/m$^2$ IV 4 h × 2 days | 94 (88) |
| | | DOX<br>MITC<br>DDP | 40 mg/m$^2$ IV bolus<br>8 mg/m$^2$ IV bolus<br>60 mg/m$^2$ IV bolus | 90 (84) |
| Santoro *et al.*,[9] 1995, EORTC | Adult STS | DOX | 75 mg/m$^2$ IV bolus | 263 (240) |
| | | DOX<br>VCR<br>CYCLO<br>DTIC | 50 mg/m$^2$ IV bolus<br>1.5 mg/m$^2$ IV bolus<br>500 mg/m$^2$ IV bolus<br>750 mg/m$^2$ IV 30 minutes | 142 (134) |
| | | DOX<br>IFOS | 50 mg/m$^2$ IV bolus<br>5 g/m$^2$ IV 24 h | 258 (231) |

NCI = National Cancer Institute; ECOG = Eastern Cooperative Oncology Group; GOG = Gynecologic Oncology Group; EORTC = European Organization for Research and Treatment of Cancer; STS = soft-tissue sarcoma; DOX = doxorubicin; STREPT = streptozotocin; VCR = vincristine; CYCLO = cyclophosphamide; DTIC = dacarbazine; VND = vindesine; IFOS = ifosfamide; MITC = mitomycin; DDP = cisplatin; IV = intravenous.

*All doses are given every 3 weeks unless stated otherwise.

†Third arm: vincristine, actinomycin D, cyclophosphamide.

**Table 2.** *Response rates and median survival times reported in randomized trials of doxorubicin chemotherapy**

| Study | Treatment | No. of evaluable patients | No. of responders (%) | Median survival (months) |
|---|---|---|---|---|
| Chang & Wiernik,[2] 1976 | DOX | 17 | 4 (24) | 10.2 |
| | DOX + STREPT | 14 | 2 (14) ($p$=NS) | 10.6 |
| Schoenfeld *et al.*,[3] 1982 | DOX | 66 | 18 (27) | 8.5 |
| | DOX + VCR + CYCLO | 70 | 13 (19) ($p$=0.03)† | 7.8 |
| Omura *et al.*,[4] 1983 | DOX | 120 | 13/80 (16) | 7.7 |
| | DOX + DTIC | 106 | 16/66 (24) ($p$=NS) | 7.3 |
| Muss *et al.*,[5] 1985 | DOX | 50 | 5/26 (19) | 11.6 |
| | DOX + CYCLO | 54 | 5/26 (19) ($p$=NS) | 10.9 |
| Borden *et al.*,[6] 1987 | DOX every 3 weeks | 94 | 17 (18) | 8.0 |
| | DOX loading every week | 88 | 15 (17) | 8.4 |
| | DOX + DTIC | 92 | 28 (30) ($p$=0.03)‡ | 8.0 |
| Borden *et al.*,[7] 1990 | DOX | 151 | 26 (17) | 9.4 |
| | DOX + VND | 147 | 26 (18) ($p$=NS) | 9.9 |
| Edmonsen *et al.*,[8] 1993 | DOX | 90 | 18 (20) | 8.4 |
| | DOX + IFOS | 88 | 30 (34) | 11.5 |
| | DOX + MITC + DDP | 84 | 27 (32) ($p$=0.03)‡ | 9.4 |
| Santoro *et al.*,[9] 1995 | DOX | 240 | 56 (23) | 12.0 |
| | DOX + VCR + CYCLO + DTIC | 134 | 38 (28) | 11.8 |
| | DOX + IFOS | 231 | 65 (28) ($p$=NS) | 12.7 |

*Abbreviations are explained in the first footnote to Table 1; NS = not significant.
† Single-agent doxorubicin better than combination chemotherapy.
‡ Doxorubicin combination chemotherapy better than single-agent doxorubicin.

*Description of studies*

There were nine single-agent doxorubicin arms (1086 total patients entered) in the eight studies. One study evaluated doxorubicin given in two different schedules.[6] Each study included an arm in which high-dose single-agent doxorubicin was given every 3 weeks. In three studies,[2,4,5] the dose was 60 mg/m$^2$; in three studies,[3,6,7] 70 mg/m$^2$; one study used a dose of 75 mg/m$^2$;[9] and a final study used a dose of 80 mg/m$^2$.[8] In one study,[6] there was an additional arm in which doxorubicin (20 mg/m$^2$ $\times$ 3) was administered as a loading dose followed by 15 mg/m$^2$ weekly.

There were 10 doxorubicin-based combination chemotherapy regimens given in eight studies (1195 total patients entered). The dose of doxorubicin in combination with other agents was 40 mg/m$^2$ in one study,[8] 50 mg/m$^2$ in two studies,[3,9] 60 mg/m$^2$ in five studies,[2,4–6,8] and 70 mg/m$^2$ in one study;[7] in each case treatment was repeated every 3 weeks. The doxorubicin-based combination chemotherapy regimens included doxorubicin with either vindesine,[7] streptozotocin,[2] or cyclophosphamide;[5] doxorubicin with ifosfamide in two studies;[8,9] doxorubicin with DTIC in two studies;[4,6] doxorubicin with mitomycin-C and cisplatin in one study;[8] doxorubicin with vincristine and cyclophosphamide in one study;[3] and doxorubicin with vincristine, cyclophosphamide and DTIC in one study.[9]

Although a few patients who had received previous chemotherapy (Table 1) were included in the earlier studies,[2–4] most patients were chemotherapy-naive when they entered these studies. Similarly, the majority had adult soft-tissue sarcoma, although a few bone sarcomas and mesotheliomas were included in three studies.[2,3,8] All the trials excluded some patients entered on study who were subsequently

**Table 3**. *Toxic effects reported in randomized trials of doxorubicin chemotherapy**

| Study | Treatment | Toxic effect | | |
|---|---|---|---|---|
| | | Nausea and vomiting | White blood cell count | Platelet count |
| Chang & Wiernik,[2] | | WBC <2000 | PLATS <100 000 | |
| 1976 | DOX | 59% mild/moderate | 9% | 3% |
| | DOX + STREPT | 100% moderate/severe | 30% | 13% |
| | | | ($p$<0.01) | ($p$<0.03) |
| Schoenfeld *et al.*,[3] | | | Haematologic | |
| 1982 | DOX | 42% moderate/severe | 17% severe | |
| | DOX + VCR + CYCLO | 60% moderate/severe | 30% severe | |
| | | ($p$=0.09) | ($p$=0.07) | |
| Omura *et al.*,[4] | | Grade 3/4 | Grade 3/4 | Grade 3/4 |
| 1983 | DOX | 2.2% | 16% | 4% |
| | DOX + DTIC | 8.5% | 35% | 13% |
| Muss *et al.*,[5] | | | WBC <2000 | PLATS <50 000 |
| 1985 | DOX | 0% severe | 10% | 0% |
| | DOX + CYCLO | 6% severe | 35% | 0% |
| Borden *et al.*,[6] | | | Haematologic | |
| 1987 | DOX every 3 weeks | 11% severe | 28% severe | |
| | DOX loading every week | 6% severe | 13% severe | |
| | DOX + DTIC | 29% severe | 29% severe | |
| | | ($p$=0.000 03) | ($p$=0.87) | |
| Borden *et al.*,[7] | | | Haematologic | |
| 1990 | DOX | 6% severe | 36% severe | |
| | DOX + VND | 3% severe | 50% severe | |
| Edmonson *et al.*,[8] | | | Haematologic | |
| 1993 | DOX | 7% severe | 53% | |
| | DOX + IFOS | 18% severe | 80% | |
| | DOX + MITC + DDP | 17% severe | 55% | |
| | | | ($p$=0.01) | |
| Santoro *et al.*,[9] | | Grade 3/4 | Grade 4 | Grade 3/4 |
| 1995 | DOX | 17% | 13% | 4% |
| | DOX + VCR + CYCLO + DTIC | 40% | 15% | 10% |
| | DOX + IFOS | NR | 32% | 6% |
| | | | ($p$<0.001) | |

*Abbreviations are explained in the first footnote to Table 1; NR = not reported; WBC = white blood cells; PLATS = platelets.

found to be ineligible and a variable number of patients were found not to be evaluable for response (Table 1).

Table 2 outlines response rates and median survival, which were consistently reported across all studies. Response rates for single-agent doxorubicin ranged between 16% and 27%. Response rates for combination chemotherapy ranged from a low of 14% for doxorubicin and streptozotocin,[2] to 34% for doxorubicin and ifosfamide.[8] Response rates were significantly better for the combination chemotherapy regimens in only two trials. In one study,[6] the combination of doxorubicin and DTIC was superior to doxorubicin ($p$=0.03), given by two different schedules, and in the second study,[8] the combination of doxorubicin and ifosfamide was superior to single-agent doxorubicin ($p$=0.03). In one study,[3] response rate was significantly better on doxorubicin compared with the combination of doxorubicin, vincristine and cyclophosphamide ($p$=0.03). None of the studies showed any significant differences in median survival time between single-agent doxorubicin and combination chemotherapy.

### *Assessment of trial quality*

Studies included in this systematic overview were published between 1976 and 1995. In general, later reports included more details about methodology, particularly statistical analysis. Five studies described a satisfactory (central office) method of randomization,[3,6–9] and four studies included an outline of statistical methodology in the 'Patient'/ 'Materials and methods' section.[4,6,7,9] However, in only two papers were accrual goals set and met.[7,9] The studies conducted by Chang & Wiernik and Muss *et al.* were of inadequate size to properly evaluate differences in response rate or survival.[2,5] Although response criteria were described or referenced in all except one study,[3] it is generally accepted that the quality of evaluation of response has improved over the past 20 years because of better imaging techniques and attention to quality-control procedures. Thus, the results reported in later studies may be more reliable. Two papers provided very limited data on toxic effects,[3,4] and only two papers provided detailed tabular reports of toxic effects seen in multiple systems.[7,9] In five studies,[4,6–8] central pathology review was performed in a majority of tumours. Some analysis of delivered dose of relevant drugs was performed in four studies.[5,6,8,9]

Overall, it was not felt that there were a sufficient number of papers, in which the quality exceeded the remainder, to justify a sensitivity analysis based on quality. However, a sensitivity analysis was performed on the four trials that included a combination of doxorubicin with at least one of the other known active agents for soft-tissue sarcoma (i.e. ifosfamide and DTIC) in their regimens.[4,6,8,9]

### *Meta-analysis results*

Data were combined for objective tumour response and overall survival. A statistical *Q*-test showed no significant numerical heterogeneity across studies for these two outcomes. The *Q*-test values were 9.45 for objective tumour response and 3.42 for overall survival. Adverse effects data were not combined, as the outcomes and measures varied greatly among studies.

*Objective tumour response.* Objective tumour response (complete and partial) data were available and consistently reported in all eight trials, providing eight comparisons with a total of 2281 patients. The trials ranged in size from 663 randomized patients,[9] to 33 randomized patients.[2] Results of pooling response data (Fig. 1) showed a slight trend favouring the combination therapy, though this did not reach statistical significance (OR, 0.79; 95% CI, 0.60–1.05; $p$=0.10). However, when the data pooling was restricted to the four trials involving combination regimens of known active agents,[4,6,8,9] this trend disappeared (OR, 0.71; 95 CI, 0.45–1.13; $p$=0.15).

*Overall survival.* Survival data were extracted directly from probability graphs for six of the eight trials, for a total of 2097 patients. In two trials, survival data either were not reported,[2] or could not be extracted.[3] Trial size ranged from 663 randomized patients,[9] to 132 randomized patients.[5] Results of pooling this outcome measure across six studies (Fig. 2) were not statistically significant (OR, 0.84; 95% CI, 0.67–1.06; $p$=0.13), and the results did not significantly change when the data were restricted to the four trials using combinations of known active agents (OR, 0.90; 95% CI, 0.69–1.20; $p$=0.48).[4,6,8,9]

*Epirubicin.* Consideration was given to broadening the guideline to include any randomized studies of single-agent anthracycline versus the same anthracycline in combination with other agents. Epirubicin has been evaluated as a single agent in two European Organization for Research and Treatment of Cancer (EORTC) randomized trials,[22,23] as well as in a number of single-arm combination chemotherapy studies. In the second EORTC study,[23] high-dose epirubicin 150 mg/m$^2$ given every 3 weeks by two different schedules produced similar response rates (14–15%) to standard-dose doxorubicin 75 mg/m$^2$ every 3 weeks (14%), with no difference in overall survival ($p$=0.89). In one randomized study from

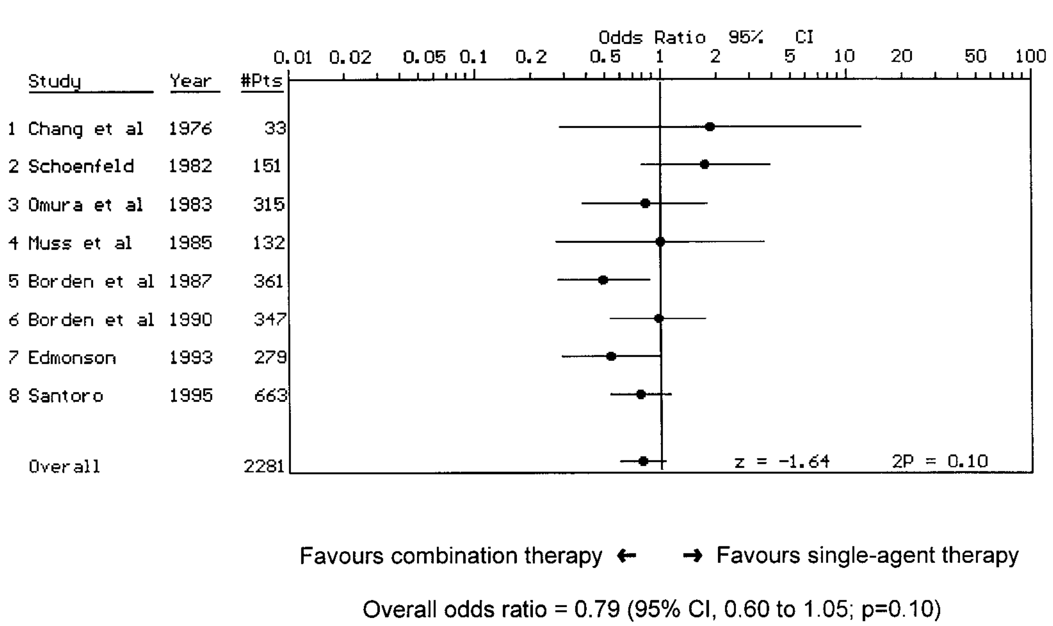

**Figure 1**. *Meta-analysis results for objective tumour response (complete and partial). Results are expressed as an OR, which is the odds of an event occurring in the experimental group (combination therapy) over the odds of an event occurring in the control group (single-agent therapy). Horizontal lines denote 95% CI. Circles represent point estimates.*

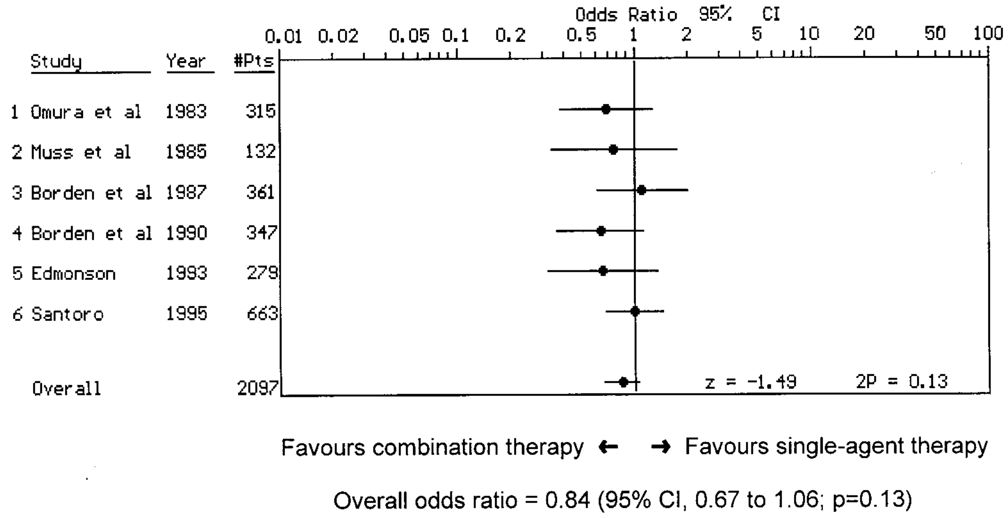

**Figure 2.** *Meta-analysis results for survival (at 2 years). Results are expressed as an OR, which is the odds of an event occurring in the experimental group (combination therapy) over the odds of an event occurring in the control group (single-agent therapy). Horizontal lines denote 95% CI. Circles represent point estimates.*

Serbia,[24] 50 patients receiving epirubicin 60 mg/m$^2$/24 h on days 1,2 and 3 (group A) were compared with 56 patients given the same dose of epirubicin + cisplatin 30 mg/m$^2$/24 h on days 2–5 (group B). The response rate was higher for group B (54% versus 29%; $p < 0.025$) and so was overall survival ($p$=0.001). However, median survival times were approximately 10 months versus 8 months, in the same range as the median survival times in studies shown in Table 2. Adding this study to the meta-analyses did not significantly alter the outcomes for response rate (OR, 0.74; 95% CI, 0.55–1.00; $p$=0.051) or survival (OR, 0.78; 95% CI, 0.60–1.03; $p$=0.078). In view of the very limited data on epirubicin, the conclusions and practice guideline are based on the doxorubicin studies.

### *Adverse effects*

Reporting of adverse effects was quite variable among the eight eligible trials. Most of the studies reported nausea/vomiting and haematologic toxic effects. As all these studies were performed before the widespread use of 5-HT$_3$ receptor antagonists, nausea and vomiting were reported frequently. As can be seen from Table 3, with the exception of the study reported by Borden *et al.*,[7] nausea and vomiting were always greater for combination regimens, often significantly so. Similarly, haematologic toxic effects were reported in different ways among studies. Leucopenia and thrombocytopenia were reported sometimes separately, sometimes in combination. In many of these studies, nadir blood counts were not necessarily performed and there may be under-reporting of haematologic toxicity. Again, it is evident from Table 3 that the haematologic toxicity of combination chemotherapy was always higher than that of single-agent doxorubicin. Neutropenic fever was not reported consistently; neither were other toxic effects, such as mucositis. Although the more recent studies did report toxic deaths,[6–9] these were uncommon across all the studies. Reporting of cardiotoxicity was highly variable and it was impossible to determine whether this was worse for single-agent or combination regimens; ultimately, it depended on the individual dose of doxorubicin received by each patient. Quality of life was not addressed in any of the studies included in this report.

### *Practitioner feedback results*

Fifty-three practitioners in Ontario, Canada, were surveyed. The sample consisted of medical oncologists, radiation oncologists, surgeons, gynaecologists and pharmacists. Of the respondents, 76% agreed with the recommendations and 72% approved the recommendations as a practice guideline. Fifty per cent of the respondents provided written comments. These comments were reviewed by the members of the Sarcoma DSG, and modifications were made to the document, where necessary, to address the comments.

There was a request for acknowledgement of trials using epirubicin alone or in combination in the palliative setting. A search was performed, and a randomized trial was added to the meta-analysis.[24] There was also a query regarding the correlation of response to chemotherapy with histological subtype of sarcoma. A paragraph was added to the 'Discussion' section to address this comment. One practitioner stated a belief that a regimen with a 30% response rate would provide a significant palliative benefit to more patients than a regimen that produced an 18% response rate. The members of the Sarcoma DSG felt that there was no evidence for this statement, as palliative benefit depends not only on response rate, but also on toxicity. No changes were made to the document. Some physicians suggested reviewing data on other agents or combinations, or producing a guideline on the general management of soft-tissue sarcoma. That was not the focus of this guideline. Consequently, no changes were made to address this comment.

### Discussion

Response rates for combination chemotherapy were significantly better than for single-agent doxorubicin in only two of the eight randomized trials. Pooling of response data showed a slight trend favouring combination chemotherapy (OR, 0.79; 95% CI, 0.60–1.05), but this did not achieve statistical significance ($p$=0.10). Similarly, combining survival data did not show a significant difference between treatment groups (OR, 0.84; 95% CI, 0.67–1.06; $p$=0.13). Although reporting of adverse effects was limited and inconsistent among trials (making pooling of data for this outcome problematic), side-effects such as nausea/vomiting and haematologic toxic effects were consistently reported as being worse with combination chemotherapy across the eight eligible studies.

A number of authors have suggested that response to chemotherapy may vary with histological subtype, although there are discrepancies between studies in identifying the most and least responsive histologies. Potential flaws of these studies include insufficient patient numbers for reliable statistical analysis and variability in pathological interpretation. The most extensive database, which has been subjected to central histopathological review, has been established by the EORTC Soft Tissue and Bone Sarcoma Group. Van Glabbeke *et al.* reported on 2185 patients with advanced soft-tissue sarcoma treated in seven clinical trials investigating the use of anthracycline-containing regimens as first-line chemotherapy.[25] Univariate analysis showed increased survival times for patients with liposarcoma and synovial sarcoma, decreased survival times for patients with malignant fibrous histiocytoma and a higher response rate for patients with liposarcoma ($p$<0.05 for all log-rank and $\chi^2$ tests). However, using multivariate analysis, the only significant influence of pathological subtype documented was that a diagnosis of liposarcoma was a favourable prognostic factor for response rate ($p$=0.006 5).

The main limitation of the present review is the fact that a number of different doxorubicin-based combination chemotherapy regimens have been compared with doxorubicin. Four of the eight studies compared combinations which included drugs considered to have limited activity as single-agent regimens in advanced soft-tissue sarcoma (i.e. vincristine, vindesine, cyclophosphamide, streptozotocin, mitomycin-C, cisplatin). However, even the four studies which used the known active agents in combination with doxorubicin (i.e. ifosfamide and DTIC) produced mixed results. Thus, the response rate for doxorubicin/DTIC was better than that for doxorubicin in one study,[6] and similar in another study.[4] Also, for doxorubicin/ifosfamide, the response rate was better than for doxorubicin alone in the study reported by Edmonson *et al.*,[8] but similar in the EORTC study reported by Santoro *et al.*[9] A meta-analysis of these four trials did not demonstrate a significant difference in response rate ($p$=0.15). The three-drug combination of doxorubicin, DTIC and ifosfamide has never been directly compared with doxorubicin alone. However, in a recent randomized study, a superior response rate was shown for the three-drug combination doxorubicin, DTIC and ifosfamide compared with the combination of doxorubicin and DTIC (32% versus 17%; $p$<0.002) but with increased myelosuppression and no improvement in overall survival.[26] Since the publication of these studies, no new active drugs have been identified in soft-tissue sarcoma.

In virtually all of the reviewed studies, the toxic effects of combination chemotherapy (particularly nausea and vomiting and myelosuppression) exceeded those of single-agent doxorubicin. It can be argued that modern anti-emetics and growth factor support might reduce or eliminate these differences, but in the setting of palliative chemotherapy, the costs of such strategies (particularly with granulocyte colony-stimulating factor) must be weighed against the expected benefits.

In the reviewed studies, 633 of 1086 patients (58%) receiving doxorubicin were given a dose of 70–75 mg/m$^2$ every 3 weeks. Toxicity data from these studies were too sparse to provide an EBR regarding dose. However, the EORTC has extensive experience of the safety and efficacy of doxorubicin 75 mg/m$^2$ every 3 weeks,[9,22,27] and this dose schedule is commonly used by sarcoma specialists throughout North America and Canada. Thus, for the palliative treatment of symptomatic locally advanced or metastatic soft-tissue sarcoma, an appropriate starting dose schedule of doxorubicin is 75 mg/m$^2$ intravenously every 3 weeks.

In summary, combinations of the known active drugs used at conventional doses can produce marginal increases in response rate in advanced/metastatic soft-tissue sarcoma, at the expense of increased adverse effects, but do not significantly increase survival rates. Thus, the results of this analysis favour the use of single-agent doxorubicin for palliative treatment of advanced/metastatic soft-tissue sarcoma.

### Practice guideline

This recommendation applies to patients with symptomatic unresectable locally advanced or metastatic soft-tissue sarcoma who are candidates for palliative chemotherapy.

- Single-agent doxorubicin is an appropriate first-line chemotherapy option for advanced or metastatic soft-tissue sarcoma. Some doxorubicin-based combination chemotherapy regimens, given in conventional doses, produce only marginal increases in response rates, at the expense of increased toxic effects, and with no improvements in overall survival.
- Future randomized clinical trials should compare new regimens, whose activity has been established in single-arm studies, with single-agent doxorubicin, and include quality of life as an outcome measure.

### Practice guideline date

Completed 30 November 1999. Updated 28 January 2000.

*Cancer Care Ontario Practice Guidelines Initiative (CCOPGI) practice guidelines are reviewed and updated regularly. Please visit the CCOPGI website at http://www.cancercare.on.ca/ccopgi/ for updates to this guideline.*

### Acknowledgements

Robert Bell, Charles Catton, Jordi Cisa, Jane Curry, Aileen Davis, C. Jay Engel, Alvaro Figueredo, Victor Fornasier, Lorraine Hands, Brian O'Sullivan, Shailendra Verma, Rebecca Wong and Caroline Zwaal also contributed to the development of this practice guideline. Please see the CCOPGI website (http://www.cancercare.on.ca/ccopgi/) for a complete list of current Sarcoma Disease Site Group members. The CCOPGI is sponsored by Cancer Care Ontario and the Ontario Ministry of Health and Long-Term Care.

### References

1 Pinedo HM, Kenis Y. Chemotherapy of advanced soft-tissue sarcomas in adults. *Cancer Treat Rev* 1997;4:67–86.
2 Chang P, Wiernik PH. Combination chemotherapy with adriamycin and streptozotocin. I. Clinical results in patients with advanced sarcoma. *Clin Pharmacol Ther* 1976;20:605–10.
3 Schoenfeld DA, Rosenbaum C, Horton J, Wolter JM, Falkson G. DeConti RC. A comparison of adriamycin versus vincristine and adriamycin, and cyclophosphamide versus vincristine, actinomycin-d and cyclophosphamide for advanced sarcoma. *Cancer* 1982;50:2757–62.
4 Omura GA, Major FJ, Blessing JA, Sedlacek TV, Thigpen JT, Creasman WT *et al.* A randomized study of adriamycin with and without dimethyl triazenoimidazole carboxamide in advanced uterine sarcomas. *Cancer* 1983;52:626–32.
5 Muss HB, Bundy B, DiSaia PJ, Homesley HD, Fowler WC, Creasman W *et al.* Treatment of recurrent or advanced uterine sarcoma: a randomized trial of doxorubicin versus doxorubicin and cyclophosphamide (a phase II trial of the Gynecologic Oncology Group). *Cancer* 1985;55:1648–53.
6 Borden EC, Amato DA, Rosenbaum C, Enterline HT, Masanori J, Shiraki MJ *et al.* Randomized comparison of three adriamycin regimens for metastatic soft tissue sarcomas. *J Clin Oncol* 1987;5:840–50.
7 Borden EC, Amato DA, Edmonson JH, Ritch PS, Shiraki M. Randomized comparison of doxorubicin and vindesine to doxorubicin for patients with metastatic soft-tissue sarcomas. *Cancer* 1990;66:862–7.
8 Edmonson JH, Ryan LM, Blum RH, Brooks JSJ, Shiraki M, Frytak S *et al.* Randomized comparison of doxorubicin alone versus ifosfamide plus doxorubicin or mitomycin, doxorubicin, and cisplatin against advanced soft tissue sarcomas. *J Clin Oncol* 1993;11:1269–75.
9 Santoro A, Tursz T, Mouridsen H, Verweij J, Steward W, Somers R *et al.* Doxorubicin versus CYVADIC versus doxorubicin plus ifosfamide in first-line treatment of advanced soft tissue sarcomas: a randomized study of the European Organization for Research and Treatment of Cancer Soft Tissue and Bone Sarcoma Group. *J Clin Oncol* 1995;13:1537–45.
10 Buesa JM, Mouridsen HT, Van Oosterom AT, Verweij J, Wagener T, Steward W *et al.* High dose DTIC in advanced soft tissue sarcomas in the adult. A phase II study of the EORTC soft tissue and bone sarcoma group. *Ann Oncol* 1991;2:307–9.
11 Bramwell VHC, Mouridsen HT, Santoro A, Blackledge G, Somers R, Verwey J *et al.* Cyclophosphamide versus ifosfamide: final report of a randomized phase II trial in adult soft tissue sarcomas. *Eur J Cancer Clin Oncol* 1987;23:311–21.
12 Stuart-Harris RC, Harper PG, Parsons CA, Kaye SB, Mooney CA, Gowing NF *et al.* High dose alkylation therapy using ifosfamide infusion with mesna in the treatment of adult advanced soft tissue sarcoma. *Cancer Chemother Pharmacol* 1983;11:69–72.
13 Antman KH, Ryan L, Elias A, Sherman D, Grier HE. Response to ifosfamide and mesna: 124 previously treated patients with metastatic or unresectable sarcoma. *J Clin Oncol* 1989;7:126–31.
14 Demetri GD, Elias AD. Results of single agent and combination chemotherapy for advanced soft tissue sarcomas: implications for decision making in the clinic. *Hematol Oncol Clin North Am* 1995;9:765–85.
15 Bramwell VH. Chemotherapy for metastatic soft tissue sarcomas—another full circle? *Br J Cancer* 1991;64:7–9.
16 Yap B-S, Benjamin RS, Burgess MA, Murphy WK, Sinkovics JG, Bodey GP. A phase II evaluation of methyl CCNU and actinomycin D in the treatment of advanced sarcomas in adults. *Cancer* 1981;47:2807–9.
17 Spielmann M, Sevin D, Le Chevalier T, Subirana R, Contesso G, Génin J *et al.* Second line treatment in advanced sarcomas with vindesine (VDS) and cisplatin (DDP) by continuous infusion (CI) [abstract]. *Proc Annu Meet Am Soc Clin Oncol* 1988;7:276. Abstract 1072.
18 Frost DB. Pulmonary metastasectomy for soft tissue sarcomas: is it justified? *J Surg Oncol* 1995;59:110–5.
19 van Geel AN, Pastorino U, Jauch KW, Judson IR, van Coevorden F, Buesa JM *et al.* Surgical treatment of lung metastases: the European Organization for Research and Treatment of Cancer, Soft Tissue and Bone Sarcoma Group study of 255 patients. *Cancer* 1996;77:675–82.
20 DerSimonian R, Laird N. Meta-analysis in clinical trials. *Control Clin Trials* 1986;7:177–88.
21 Browman GP, Levine MN, Mohide EA, Hayward RSA, Pritchard KI, Gafni A *et al.* The practice guidelines development cycle: a conceptual tool for practice guidelines development and implementation. *J Clin Oncol* 1995;13:502–12.
22 Mouridsen HT, Bastholt L, Somers R, Santoro A, Bramwell V, Mulder JH *et al.* Adriamycin versus epirubicin in advanced soft tissue sarcomas. A randomized phase II/phase III study of the EORTC Soft Tissue and Bone Sarcoma Group. *Eur J Cancer Clin Oncol* 1987;23:1477–83.
23 Nielsen OS, Dombernowsky P, Mouridsen H, Crowther D, Verweij J, Buesa J *et al.* High-dose epirubicin is not an alternative to standard-dose doxorubicin in the treatment of advanced soft tissue sarcomas. A study of the EORTC soft tissue and bone sarcoma group. *Br J Cancer* 1998;78:1634–9.

24 Jelic S, Kovcin V, Milanovic N, Babovic N, Kreacic M, Ristovic Z *et al*. Randomised study of high-dose epirubicin versus high-dose epirubicin–cisplatin chemotherapy for advanced soft tissue sarcoma. *Eur J Cancer* 1997;33:220–5.

25 Van Glabbeke M, van Oosterom A, Oosterhuis JW, Mouridsen H, Crowther D, Somers R *et al*. Prognostic factors for the outcome of chemotherapy in advanced soft tissue sarcoma: an analysis of 2185 patients treated with anthracycline-containing first-line regimens—a European Organization for Research and Treatment of Cancer Soft Tissue and Bone Sarcoma Group Study. *J Clin Oncol* 1999;17:150–7.

26 Antman K, Crowley J, Balcerzak SP, Rivkin SE, Weiss GR, Elias A *et al*. An intergroup phase III randomized study of doxorubicin and dacarbazine with or without ifosfamide and mesna in advanced soft tissue and bone sarcomas. *J Clin Oncol* 1993;11:1276–85.

27 Bramwell VHC, Mouridsen HT, Mulder JH, Somers R, Van Oosterom AT, Santoro A *et al*. Carminomycin vs adriamycin in advanced soft tissue sarcomas: an EORTC randomised phase II study. *Eur J Cancer Clin Oncol* 1983;19:1097–104.
